# Essential oil-derived compounds target core fatigue-related genes: A network pharmacology and molecular Docking approach

**DOI:** 10.1371/journal.pone.0314125

**Published:** 2025-05-28

**Authors:** Gyaltsen Dakpa, Yu-Ting Chiang, Li-Yin Lin, Nai-Wen Tsao, Chung-Hsuan Wang, Horacio Pérez-Sánchez, Jorge Ricardo Alonso Fernández, Sheng-Yang Wang

**Affiliations:** 1 Molecular and Biological Agricultural Sciences Program, Taiwan International Graduate Program, Academia Sinica, Taipei, Taiwan; 2 Graduate Institute of Biotechnology, National Chung-Hsing University, Taichung, Taiwan; 3 Liyu International Co., Ltd, Taichung, Taiwan; 4 Special Crop and Metabolome Discipline Cluster, Academy of Circle Economy, National Chung Hsing University, Taichung, Taiwan.; 5 Department of Forestry, National Chung-Hsing University, Taichung, Taiwan; 6 Structural Bioinformatics and High-Performance Computing (BIO-HPC), Campus de los Jerónimos, Universidad Católica de Murcia (UCAM), Guadalupe, Murcia, España (Spain),; 7 Agricultural Biotechnology Research Center, Academia Sinica, Taipei, Taiwan; Kwara State University, NIGERIA

## Abstract

Fatigue is a widespread condition associated with various health issues, yet identifying specific bioactive compounds for its management remains challenging. This study integrates network pharmacology and molecular docking to uncover essential oil-derived compounds with potential antifatigue properties by targeting key genes and molecular pathways. A comprehensive analysis of 872 essential oil compounds was conducted using PubChem, with target prediction via SwissTargetPrediction. The protein-protein interaction (PPI) network and KEGG pathway analysis identified core fatigue-related targets, including ALB, BCL2, EGFR, IL-6, and STAT3, in metabolic dysregulation and inflammatory responses linked to fatigue. Molecular docking exhibits strong binding affinity between key compounds such as Calamenene, T-cadinol, and Bornyl acetate and core targets, suggesting their potential antifatigue effects. However, ADMET analysis confirmed T-cadinol’s drug-likeness, suggesting good bioavailability and minimal toxicity risks. Thus, molecular docking revealed high binding affinity, which was further validated through a 100 ns MD simulation and demonstrated stable interactions with low root mean square deviation (RMSD). Additionally, hydrogen bond analysis confirmed that T-cadinol maintained consistent interactions with key residues such as Thr-790 in EGFR, Arg-222 in ALB, and Arg-104 in IL-6, indicating strong binding stability. While this study provides valuable computational insights, further in vitro and in vivo validation is necessary to confirm these findings and explore potential therapeutic applications.

## Introduction

Fatigue is a complex symptom experienced by healthy individuals and individuals with acute and chronic medical conditions. It can lead to psychosocial problems, depression, anxiety disorders, sleep disorders, and sleep-related respiratory disorders [1,2]. Fatigue is highly associated with excess mortality in the general population. It can be caused by neoplasms or infections such as human immunodeficiency virus (HIV) or tuberculosis (TB) [3]. SARS-CoV-2 infection has also contributed to post-acute sequelae of COVID-19 (PASC), including post-exertional malaise, brain fog, dizziness, and fatigue [4]. The World Health Organization (WHO) has classified fatigue as one of the significant risk factors for human life and health, as it can also be the sequelae of multiple complex diseases [5]. Most cancer patients suffer from severe fatigue during chemotherapy or radiotherapy, which is highly associated with the central nervous system (CNS). There is ample evidence explaining the mechanisms of fatigue, including skeletal muscular and mitochondrial dysfunction, peripheral immune activation and inflammation dysfunction, and CNS disorders. Mitochondrial DNA (mtDNA), mitochondrial structure, oxidative pressure, pro-inflammatory and anti-inflammatory cytokines in the peripheral system, even in the CNS, and Adenosine triphosphate (ATP) all play significant roles in the induction of fatigue.

Additionally, neuropeptide, neurotransmitter, and hypothalamic-pituitary-adrenal (HPA) axis dysfunction in CNS tend to amplify the sense of fatigue [6,7]. The multifactorial causes of fatigue discussed above and current approved therapeutics for fatigue management include modafinil, amantadine, and various antidepressants that target the central nervous system to improve alertness and mood. Despite their clinical use, these pharmacological treatments often have limitations and side effects. In contrast, essential oils have a long history in folklore medicine as natural antifatigue remedies. For example, peppermint, rosemary, and thyme oils have traditionally been employed to boost energy levels, enhance mood, and alleviate feelings of tiredness. This traditional use—supported by emerging preclinical evidence underscores the potential of essential oil-derived compounds as alternative or complementary therapies for fatigue, warranting further investigation into their molecular mechanisms and therapeutic efficacy. Thus, finding potential anti-fatigue drugs or formulations with definite effectiveness and fewer side effects is essential.

Natural compounds have been powerfully demonstrated to exert significant anti-fatigue effects, including *Astragalus membranaceus* [8] and *Cordyceps militaris* [9]. In China, over 1159 anti-fatigue nutraceuticals were listed as of 2019. Many of these natural compounds, such as *Cornu Cervi pantotrichum* [10] and *Gynostemma pentaphyllum* (Thunb.) Makino [11] and *Portulaca oleracea* [12] have been shown to improve animal exercise capacities, such as prolonging swimming and running times or increasing forelimb grip strength. This indicates that natural compounds derived from plants and fungi with anti-fatigue effects are diverse, making them worthy of development and utilization as novel anti-fatigue drugs. However, the issues of complex composition and unclear product placement are ubiquitous.

Essential oils are complex mixtures of aromatic substances derived from plants. The composition of each essential oil depends on factors such as the plant family, the specific part of the plant from which it is extracted, the soil where it grows, and even the harvest timing [13]. The five major constituents of essential oil plants, classified by their source, include *Rosmarinus officinalis* [14], *Thymus vulgaris* [15], Peppermint [16], *Salvia officinalis* [17], and *Zingiber officinale* [18]. These oils have been applied for various biological activities, such as antiparasitic, antifungal, antibacterial, antiviral, antioxidant, anti-inflammatory, anticancer, antiaging, and neuroprotective properties [19]. Essential oils can be extracted from different parts of plants, including leaves, bark, flowers, buds, seeds, and peels. They are concentrated hydrophobic mixtures of hydrocarbon volatile compounds that quickly evaporate at room temperature, allowing inhalation *via* the olfactory system [20]. The components are detected by olfactory receptors in the nasal olfactory epithelium, stimulation of olfactory nerves, and transmission of a signal to the CNS, including the limbic system and hypothalamus, which modulates human behavior and body function [21]. Fatigue is primarily linked to CNS disorder, and essential oils influence the body’s response due to their interaction with the nervous system. However, essential oil-derived compounds remain underexplored in fatigue management due to their complex chemical composition, limited target identification, and insufficient systematic pharmacological validation. Essential oils contain hundreds of bioactive molecules, making it challenging to identify specific compounds responsible for their effects, and most studies focus on whole extracts rather than individual components. Furthermore, the mechanisms by which these compounds influence fatigue-related pathways remain unclear, as traditional approaches frequently lack computational support validation. Therefore, our study integrates network pharmacology, molecular docking, and molecular dynamics (MD) simulations to systematically identify, validate, and predict essential oil compounds’ anti-fatigue potential, providing strong computational evidence for their therapeutic potential.

Network pharmacology and molecular docking are widely recognized for their efficiency and scalability, enabling rapid analysis of vast datasets. These techniques substantially cut down the time and cost of drug discovery while enhancing the predictive accuracy of ligand-target protein binding interactions [22]. Network pharmacology provides a novel paradigm to uncover and visualize the underlying interaction networks of natural compounds against multifactorial diseases [23]. An integrated network of pharmacology and molecular docking contributes tremendously to drug discovery. For instance, Mahuang FuziXixin Decoction (MFXD) has been studied to elucidate the active components, potential targets, and molecular mechanisms against lung cancer, offering a promising strategy for exploring new roles of natural compounds and understanding the disease mechanism, such as berberine’s regulating of autophagy in breast cancer [24]. This study presents a novel approach by integrating network pharmacology and molecular docking to identify essential oil-derived compounds with potential antifatigue properties systematically. Unlike conventional pharmacological screening, network pharmacology enables the identification of multiple interconnected targets and pathways, providing a systems-level understanding of fatigue mechanisms. Molecular docking further validates the binding interactions between bioactive compounds and core fatigue-related proteins (ALB, BCL2, EGFR, IL-6, and STAT3), ensuring a more targeted and mechanistic approach to drug discovery. Additionally, molecular dynamics (MD) simulations strengthen these findings by evaluating the stability and interaction dynamics of the most promising compound, T-cadinol, over time. Cytoscape 3.10.1 implies constructing components and corresponding target networks integrated with the STRING using the protein-protein interaction (PPI) network [25]. CB-Dock2 was employed for docking, achieving a predicted success rate of over 85% and outperforming other tools such as FitDock, MTiAutoDock, SwissDock, and COACH-Dn [26]. The Protein-Ligand Interaction Profiler (PLIP) was utilized to understand the detailed interaction between the targeted protein and ligand [27]. Finally, the PyMOL molecular graphics system was used to visualize detailed chemical interactions in the ligand-protein complex. By combining these advanced in silico methodologies, this study represents one of the first efforts to apply network pharmacology, molecular docking, and MD simulation in a structured workflow to identify precise anti-fatigue compounds from essential oils. This approach accelerates the discovery of natural anti-fatigue agents and provides a computational framework for future in vitro and in vivo validation, potentially leading to novel fatigue therapeutics.

## Materials and methods

### Selection of essential oil plants and compounds

To systematically identify potential anti-fatigue compounds, we selected 5 essential oil-producing plants*: Rosmarinus officinalis* [14], *Thymus vulgaris* [15], Peppermint (Mentha × Piperita) [16], *Salvia officinalis* [17], and *Zingiber officinale* [18]. These plants were chosen based on their extensive traditional use in fatigue management, previous pharmacological studies highlighting their bioactive properties, and their rich composition of volatile compounds known for neurological and metabolic effects. Each of these plants has demonstrated biological activities such as anti-inflammatory, antioxidant, neuroprotective, and cognitive-enhancing effects, which are relevant to fatigue-related mechanisms. Additionally, their essential oils are widely used in aromatherapy and herbal medicine, supporting their potential role in alleviating fatigue symptoms. A total of 872 essential oil-derived compounds were included in this study, selected from literature-reported bioactive constituents and public chemical databases such as PubChem. The selection criteria were based on compound availability, documented pharmacological activity, and relevance to fatigue-related pathways from five primary sources: *Rosmarinus officinalis* [14], *Thymus vulgaris* [15], Peppermint [16], *Salvia officinalis* [17], and *Zingiber officinale* [18], as shown in S1-S3 Table. The structure of most active compounds was obtained from PubChem, https://pubchem.ncbi.nlm.nih.gov, the world’s most extensive collection of freely accessible chemical information, by entering their common name. The remaining compounds are drawn using ChemSpace https://chem-space.com.

### Screening for potential target proteins for essential oil compounds against fatigue

Putative targets of all selected compounds are predicted using the SwissTargetPrediction databases [28], with *Homo sapiens* selected one by one. The identified target proteins are exported into an Excel sheet. All potential essential oil targets were merged, and duplicates were removed to finalize the essential oil targets. Each compound from five primary sources has common and unique active details compounds and targets, as shown in S1 Table. For the convenience of readers, active compounds and targets for each herbal medicine were separately imported into the EVenn [29], based on Jvenn, Python, to plot the intersection of compounds and targets in the Venn diagram.

### Unveiling targets for anti-fatigue

An extensive search was conducted in multiple databases, including Online Mendelian Inheritance in Man (OMIM) [30], DisGeNET 7.0 [31], and GeneCards v5.13 [32] using the keywords ‘fatigue’ and ‘anti-fatigue.’ This resulted in an initial set of 3,000 + potential fatigue-related targets. To refine this set, we identified overlapping targets with the predicted essential oil targets from SwissTargetPrediction, leading to a final list of 115 high-confidence targets. Additionally, these databases provided concise genomic information and functional annotation of known human genes. Duplicated genes were discarded, and UniProtKB [33] was used to standardize the name of the target gene, selected as “*Homo sapiens”* as the organism. A Venn plot was created using Evenn to identify common targets for further analysis. The shared targets identified were evaluated as potential sites for essential oil compounds to combat fatigue (Fig.2).

**Fig 1:**
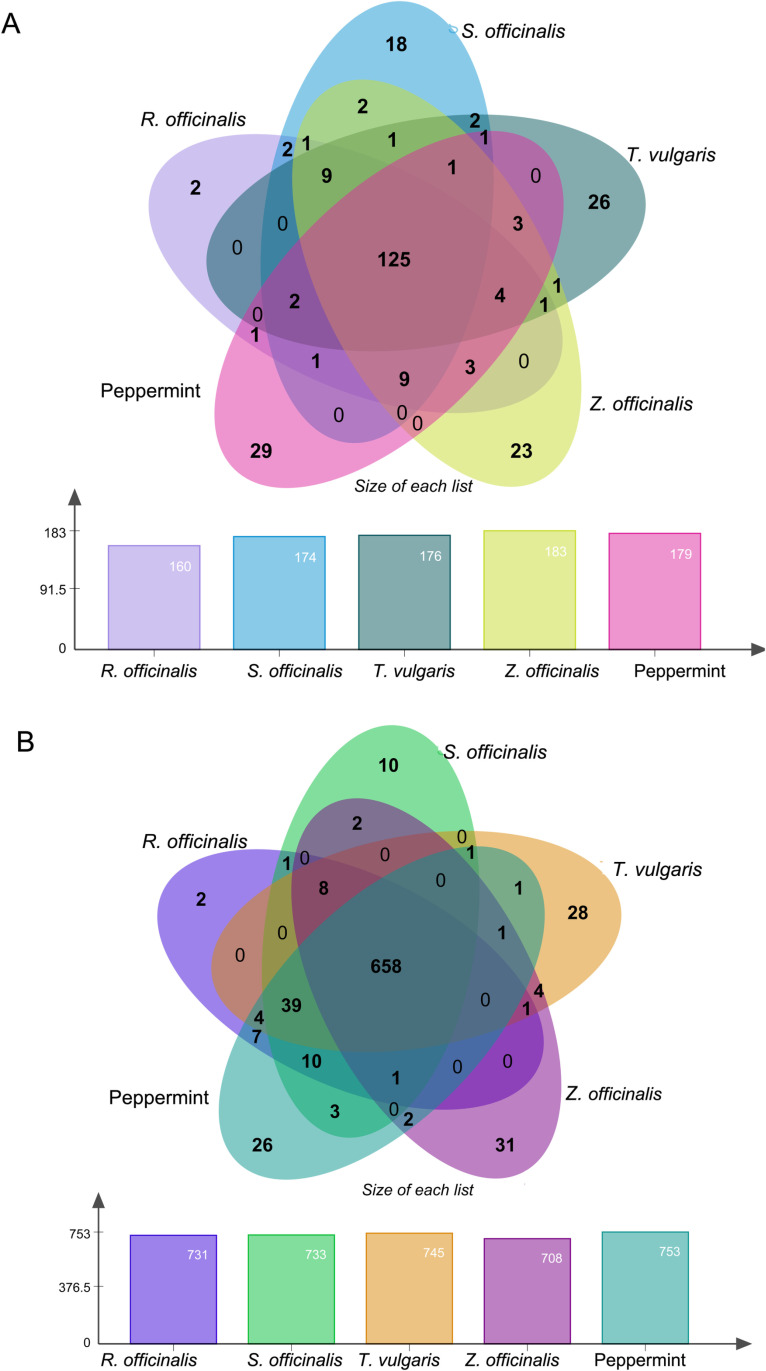
Intersection and distinction of essential compounds and targets from 5 primary sources. **(A)** The active compounds are derived from the 5 primary plant sources. These are obtained from extensive literature research, in which 125 compounds intersect each other, plotted by the Venn. (Purple for *R. officinalis* contains 160 compounds; light blue for *S. officinalis* has 733 compounds; dark green for *T. vulgaris* contains 176 compounds; light yellow for *Z. officinalis* has 183 compounds; and Pink for Peppermint contains 179 compounds. **(B)** UniProt and SwissTarget Prediction-predicted target proteins and 658 actives are intersected using the Jvenn Python to plot the intersection of targets in which Purple represents *R. officinalis*, Green indicates S. officinalis, Orange represents ***T.*** *Vulgari*s, Orchid represents *Z. officinalis,* and Turquoise represents Peppermint*.*

### Development of the essential oil plants and target network

Degree centrality, a measure of a node’s connectivity within the network, was used to prioritize compounds with extensive interactions with fatigue-related targets. A threshold was applied, selecting compounds with a degree centrality value ≥2 standard deviations above the mean of all compounds in the network to ensure statistical significance. We confirmed that the top-ranked compounds were consistently prioritized across both measures. This approach allowed us to refine our selection to 10 key compounds with the highest network influence, ensuring robust biological relevance for subsequent molecular docking and ADMET evaluation. The network mapping software Cytoscape 3.10.1 was used to construct networks for the compounds and their targets to understand the complex interactions between essential oil derivative compounds and their corresponding targets. In the network, each node represents a target, gene, molecule, or protein, while connections between nodes represent the interactions among these elements. A node’s “degree” value represents the number of connections it has within the network. Crucial high-degree nodes were identified using the “Analyze Network” function, determining the essential anti-fatigue compounds. A network analyzer tool was employed to calculate degree, a topological property that reveals the importance of compounds, target genes, and pathways in the network diagram. Target genes with the highest degree of connectivity were considered significant for anti-fatigue effects.

### Establishment of the protein-protein interaction (PPI) network

To construct a PPI network for essential oil activity against fatigue, the STRING 11.5 database [34] was utilized. Due to the large data size, not all compounds could be included initially. Cytoscape 3.10.1 was employed to create the initial network. Only target genes with the highest connectivity degrees were considered antifatigue and included in the PPI network. The biological species was set to “Homo sapiens,” with a minimum interaction threshold of “0.5,” while other parameters remained at their default settings to generate the PPI network. Visualization and analysis of the PPI network were then performed using Cytoscape 3.10.1. Finally, the top-ranked targets by network degree were identified as the critical anti-fatigue targets.

### Pathway and functional enrichment analysis

To assess potential biases in the filtering process, we performed Gene Ontology (GO) and KEGG pathway enrichment analysis on the complete set of 3,000 + targets and the filtered set of 115 targets. The study was conducted using the DAVID database [35] (Homo sapiens), with “Gene List” selected as the list type. Pathways and biological processes were considered significantly enriched if p < 0.01 and enrichment factor > 1.5. The data were then sorted based on gene count, independent of their direct association with antifatigue mechanisms. For further analysis, we selected the top GO terms from the Biological Process (BP), Cellular Component (CC), and Molecular Function (MF) categories, as well as the top 15 KEGG pathways relevant to fatigue-related biological functions. All genes were subsequently filtered and prioritized using Cytoscape, applying degree centrality as a ranking metric—a network connectivity measure highlighting compounds with extensive interactions with fatigue-related targets. A threshold was applied, selecting compounds with degree centrality values exceeding two standard deviations above the mean, ensuring statistical robustness. The bioinformatics online platform was used for data visualization [36].

### ADMET (absorption, distribution, metabolism, excretion, and toxicity) analysis of critical components for molecular docking

The potential anti-fatigue compounds, represented in SMILES format, were imported into the ADMETIab 2.0 database [37]. Using the ADMET Screening function, we obtained various physicochemical properties, including the octanol/water partition coefficient (logP), the number of hydrogen bond donors (HBD), and the number of hydrogen bond acceptors (HBA). To assess the drug-likeness of these ligands, we applied Lipinski’s rule of five parameters, which include a molecular weight of less than 500 Da, no more than five hydrogen bond donors, fewer than 5 hydrogen bond acceptors, and a ClogP value between 2 and 5. Additionally, plasma protein binding (PPB), cytochrome P450 (CYP) enzyme interactions, and toxicity predictions (hepatotoxicity, mutagenicity, and hERG inhibition) are included to assess drug metabolism and safety. This evaluation was performed using the admetSAR server2 web tool.

### Molecular docking of potential active compounds with potential target protein

To predict the binding interactions between essential oil-derived compounds and fatigue-related targets, we used a highly advanced molecular docking tool called CB-Dock2 [26], automatically detecting potential binding pockets and optimizing docking poses. The crystal structure of the potential antifatigue protein was obtained from the Protein Data Bank. PyMOL was used to streamline the docking process to remove bound ligands, surrounding water molecules, native zinc ions, extra chains with missing hydrogen atoms, and charge states. Only one chain was selected for docking. The docking process involves four steps: (i) the target protein’s PDB file and the active compounds’ SDF files were uploaded separately for data input. (ii) During data processing, the fixed chain was prepared by removing water and heterogeneous groups and adding hydrogen using the PyMOL Molecular Graphic System. The initial ligand conformation was checked, and the 3D conformation was generated to fit the target protein. Hydrogen and charges were added to ligands with FP2 greater than 0.4. (iii) The search for protein and ligand templates with RMSD less than 2.5 Å and FP2 greater than 0.4 Å resulted in five templates with the best FP2 similarities, producing the optimal binding pose for each site. (iv) The best cavities in the targeted protein for the selected ligand were identified based on binding affinity energy. CB-Dock2 determined the binding affinity energy (kcal/mol) of the tested compounds with the target protein using docking scores and selected the highest binding energy for further analysis [38]. The 3D coordinates of the protein and ligand volumes (Å³) and grid boxes were generated automatically, including center size (x, y, z) and docking size (x, y, z). Ligand-residue contacts were summarized (see Table 2). This method improves accuracy by integrating template-based predictions with flexible docking simulations, reducing the risk of misidentifying non-specific binding sites [39]. Additionally, a heatmap was plotted using Heatmapper [40].

**Table 1:** ADMET properties of selected essential oil-derived compounds for anti-fatigue drug development.

Compounds /Pubhem ID	MW (g/mol)	LogP ≤4.15	HBA	HBD	LopS	BBB	TPSA (Å^2^)	PPB %	CYP Metabolism	Toxicity Alerts
4-terpineol /11230	154.253	2.65	1	1	-1.8	0.99	20.23	75.60	Non-Inhibitor	No hepatotoxicity No mutagenicity No hERG risk
Thymol /6989	150.221	3.41	1	1	-2.77	0.916	20.23	72.40	CYP2D6 Inhibitor	Possible hepatotoxicity No mutagenicity No hERG risk
Terpinolene /11463	136.238	4.67	0	0	-3.88	0.964	0	80.30	CYP3A4 Substrate	No hepatotoxicity No mutagenicity, Possible hERG risk
Bornyl acetate /93009	196.29	3.88	2	0	-3.5	0.815	26.3	82.10	Non-Inhibitor	No hepatotoxicity No mutagenicity No hERG risk
Isomenthone /70962	154.253	3.77	1		0	0.986	17.07	78.50	CYP2C9 Inhibitor	No hepatotoxicity Possible mutagenicity No hERG risk
⍺-copaene /12303902	204.357	5.47	0	0	-5.57	0.977	0	85.30	CYP3A4 Substrate	Possible hepatotoxicity No mutagenicity No hERG risk
Calamenene /6429077	202.341	5.55	0	0	-5.7	0.978	0	88.70%	CYP1A2 Inhibitor	High hepatotoxicity No mutagenicity No hERG risk
Compounds /Pubhem ID	MW (g/mol)	LogP ≤4.15	HBA	HBD	LopS	BBB	TPSA (Å2)	PPB %	CYP Metabolism	Toxicity Alerts
T-cadinol /160799	222.372	4.95	1	1	-4.58	0.97	20.23	80.10	CYP2C9 Inhibitor	No hepatotoxicity No mutagenicity No hERG risk
Linalyl acetate /8294	196.29	3.97	2	0	-3.29	0.98	26.3	74.80	Non-Inhibitor	No hepatotoxicity No mutagenicity No hERG risk
Cuparene /86895	202.341	5.25	0	0	-5	0.98	0	87.50	Non-Inhibitor	No hepatotoxicity, No mutagenicity Possible hERG risk

Note: Lipinski Rule: MW ≤ 500; ClogP = 2–5; HBA ≤ 10; HBD ≤ 5 are acceptable.

**Table 2:** Docking scores with essential oil-derived compounds against selected target proteins.

Protein (PDB ID)	CTNNB1 (1G3J)		ALB (7VR0)		BCL2 (6GL8)		EGFR (4I23)		ESR1 (6VPF)		IL-6 (1ALU)		PPARG6 (6TSG)		PTSGS2 (5KIR)		STAT3 (6TLC)		TNF (1TNF)	
4-terpineol (kcal/mol)	-4.3 ± 0.2		-5.8 ± 0.2		-5.4 ± 0.3		-5.6 ± 0.3		-6.2 ± 0.3		-5.2 ± 0.2		-5.5 ± 0.2		-6.7 ± 0.2		-4.5 ± 0.3		-4.5 ± 0.3	
α-copaene (kcal/mol)	-4.6 ± 0.3		-7.8 ± 0.3		-7.4 ± 0.2		-6.8 ± 0.2		-7.8 ± 0.2		-5.9 ± 0.3		-7.0 ± 0.3		-7.4 ± 0.3		-5.0 ± 0.2		-5.6 ± 0.2	
Bornyl acetate (kcal/mol)	-3.6 ± 0.2		-6.0 ± 0.1		-5.9 ± 0.2		-5.7 ± 0.1		-6.8 ± 0.2		-6.0 ± 0.2		-6.0 ± 0.2		-5.4 ± 0.2		-4.4 ± 0.3		-5.0 ± 0.2	
Calamenene (kcal/mol)	-5.8 ± 0.3		-7.8 ± 0.3		-7.5 ± 0.3		-7.1 ± 0.3		-7.9 ± 0.3		-6.4 ± 0.3		-6.9 ± 0.3		-7.6 ± 0.3		-6.4 ± 0.3		-5.6 ± 0.3	
Cuparene (kcal/mol)	-4.1 ± 0.2		-7.2 ± 0.2		-6.5 ± 0.2		-7.1 ± 0.2		-8.2 ± 0.2		-6.5 ± 0.2		-6.5 ± 0.2		-7.5 ± 0.2		-5.0 ± 0.2		-5.5 ± 0.2	
Isomenthone (kcal/mol)	-4.1 ± 0.3		-6.0 ± 0.3		-5.3 ± 0.3		-5.6 ± 0.3		-6.6 ± 0.3		-4.9 ± 0.3		-6.3 ± 0.3		-6.6 ± 0.3		-5.0 ± 0.3		-4.4 ± 0.3	
Linalyl acetate (kcal/mol)	-3.9 ± 0.2		-6.1 ± 0.2		-5.3 ± 0.2		-5.7 ± 0.2		-6.2 ± 0.2		-4.9 ± 0.2		-5.7 ± 0.2		-6.2 ± 0.2		-4.2 ± 0.2		-4.6 ± 0.2	
T-cadinol (kcal/mol)	-4.3 ± 0.3		-7.0 ± 0.3		-6.9 ± 0.3		-6.7 ± 0.3		-8.1 ± 0.3		-6.1 ± 0.3		-7.0 ± 0.3		-8.1 ± 0.3		-5.9 ± 0.3		-5.2 ± 0.3	
Terpinolene (kcal/mol)	-4.1 ± 0.2		-6.2 ± 0.2		-5.5 ± 0.2		-5.4 ± 0.2		-6.4 ± 0.2		-4.7 ± 0.2		-5.3 ± 0.2		-6.7 ± 0.2		-4.7 ± 0.2		-4.4 ± 0.2	
Thymol (kcal/mol)	-4.2 ± 0.3		-6.1 ± 0.3		-5.5 ± 0.3		-5.7 ± 0.3		-6.0 ± 0.3		-5.0 ± 0.3		-5.7 ± 0.3		-6.4 ± 0.3		-5.0 ± 0.3		-4.5 ± 0.3	
Reference Ligand (kcal/mol)	-8.0 ± 0.2 XAV939		-8.2 ± 0.2 Warfarin		-9.0 ± 0.1 Venetoclax		-8.5 ± 0.2 Gefitinib		-8.7 ± 0.2 Tamoxifen		-7.2 ± 0.1 Tocilizumab		-8.4 ± 0.2 Rosiglitazone		-8.0 ± 0.2 Celecoxib		-7.8 ± 0.2 Niclosamide		-7.5 ± 0.2 Etanercept	
Docking Parameter																				
Ligand-protein complex		CTNNB1 /1G3J		ALB /7VR0		BCL2 /6GL8		EGFR /4I23		ESR1 /6VPF		IL-6 /1ALU		PPARG6 /6TSG		PTSGS2 /5KIR		STAT3 /6TLC		TNF /1TNF
Cavity Volume (Å^3^)		222		6694		323		293		704		166		218		14625		485		106
Center (X, y, z)		53, 125, 32		–2,4, 15		12, 2, 20		0, -54, -23		11, -9, 69		14, -31, 0		–6,–5, –38		19, 12, 37		–27, 7, –15		33, 47, 57
Docking Size (x, y, z)		18, 18, 18		35, 18, 26		18, 18, 18		18, 18, 18		18, 18, 24		18, 18, 18		18, 18, 18		35, 35, 34		18, 18, 18		18, 18, 18

Note: For the center coordinates (x, y, z) and docking size (x, y, z), we used CB-Dock2, which provides the three-dimensional coordinates of the protein’s region in two key forms. The center size (x, y, z) defines the central point within the receptor’s binding site where the ligand is positioned, while the docking size (x, y, z) represents the coordinates of the ligand bound to the protein. The binding affinities (kcal/mol, Mean ± SD) of selected essential oil-derived compounds against key protein targets involved in fatigue mechanisms. The docking scores represent the average of three independent docking runs, with standard deviation (SD) indicating variability. Reference ligands for each target are included for comparative evaluation.

### Verification of the molecular docking using the PLIP

To ensure accurate results, the docking protocol was validated by reproducing the co-crystallized ligand’s binding pose and molecular interactions within the active site of the experimentally crystallized protein structure. Protein-Ligand Interaction Profiler (PLIP) was employed to identify non-covalent interactions between biological macromolecules and their ligands, offering pi-cation interactions, salt bridges, water bridges, and halogen bonds, including pi-cation interactions, salt bridges, water bridges, and halogen bonds. These results were visualized using PyMOL. Hydrogen atoms were added to the ligand using PyMOL to facilitate interactions. PLIP was run for detailed interactions, including pi-cation interactions, salt bridges, hydrogen bonds, and hydrophobic and halogen bonds. The interactions between the protein and ligand and the detailed protein-ligand complex were visualized and analyzed using PyMOL.

### Validation of the molecular docking using molecular dynamic (MD) simulation

Molecular docking is inherently predictive and may generate false positives, where high docking scores do not always translate to biological activity. To mitigate this, we performed molecular dynamics (MD) simulations on T-cadinol, the most promising compound, to validate its binding stability over 100 ns. Conversely, false negatives may arise due to docking algorithms treating proteins as rigid structures, which may overlook conformational changes essential for ligand binding. We conducted molecular dynamics (MD) simulations using Maestro-Desmond, implemented by Schrödinger, to gain deeper insights into the molecular docking results. After the docking calculations, the resulting ligand-protein complexes were utilized as initial structures for the MD simulations. For preprocessing, the protein preparation wizard in Maestro was employed to add missing hydrogen atoms and side chains. The complexes were charged using the OPLS3e force field through Maestro’s system builder [41]. To ensure an accurate representation of the system, the ligand-protein complexes were immersed in a simple point charge (SPC) water box, maintaining a buffer of 10 Å beyond the complex’s atoms to prevent the protein from leaving the water box [42]. To mimic physiological conditions, 0.15 M NaCl was added to the solution. The required amounts of Na + and Cl− ions were automatically determined. Three additional Na+ ions were incorporated for Citronellol and Limonene to neutralize the system, while four Na+ ions were added in all other cases. The MD simulations were carried out under constant temperature (300 K) and pressure (1 atm) conditions, utilizing the NPT ensemble to maintain a steady number of particles throughout the simulation. The OPLS3e force field was applied to all simulations. Each protein-ligand complex was simulated for 100 ns, with data recorded every 4.8 ps. Plots and figures illustrating the simulations were generated using Maestro’s Desmond simulation interaction diagram tool.

## Results

### Intersections and distinctions in essential oil compounds and targets from five medicinal plants for anti-fatigues

A total of 872 bioactive compounds were identified from essential oils derived from five medicinal plants: Rosmarinus officinalis, Thymus vulgaris, Peppermint, Salvia officinalis, and Zingiber officinale. These plants are widely known for their anti-inflammatory, antioxidant, neuroprotective, and immune-modulating properties, making them strong candidates for fatigue management. We analyzed compound overlap across these plant sources to investigate the phytochemical diversity and potential synergistic effects (Fig 1A). Among the 872 compounds, 160 were identified in *R. officinalis,* 176 in *T. vulgaris,* 174 in *S. officinalis*, 183 in *Z. officinale*, and 179 in Peppermint. Interestingly, 125 compounds were shared among all five sources, suggesting a common pharmacophore framework contributing to their observed biological activities. The presence of key shared bioactive, including 4-terpineol, thymol, linalool, terpinolene, and α-pinene, indicates their potential role in modulating inflammatory pathways (NF-κB, IL-6, and TNF-α signaling), reducing oxidative stress (via Nrf2 activation), and regulating neurotransmitter systems (GABAergic and dopaminergic pathways). Interestingly, 125 compounds were shared among all sources, as shown in Fig 1B. This suggests that the analysis of compound intersections and non-intersections across different plant sources reveals potent biochemical synergism, which might explain the traditional use of these herbs in complementary and alternative medicine. The UniProt and SwissTargetPrediction databases were employed to predict the potential target proteins, resulting in 731, 733, 745, 708, and 753 potential target proteins from *R. officinalis, S. officinalis, T. vulgaris, Z. officinalis,* and Peppermint, respectively (Fig 1B). A total of 658 potential proteins were shared among essential oil plants. However, 2 unique proteins were identified in *R. officinalis,* 10 in *S. officinalis,* 28 in *T. vulgaris,* 31 in *Z. officinalis,* and 26 in Peppermint (Fig 1B). These findings indicate that despite the significant intersections, a subset of compounds from each source remained unique, contributing to their distinct therapeutic properties. This led us to analyze further to understand their effects better.

### Identifying and analyzing fatigue-related targets of essential oil compounds using GeneCards, OMIM, DisGeNET, and Cytoscape

**Fig 2:**
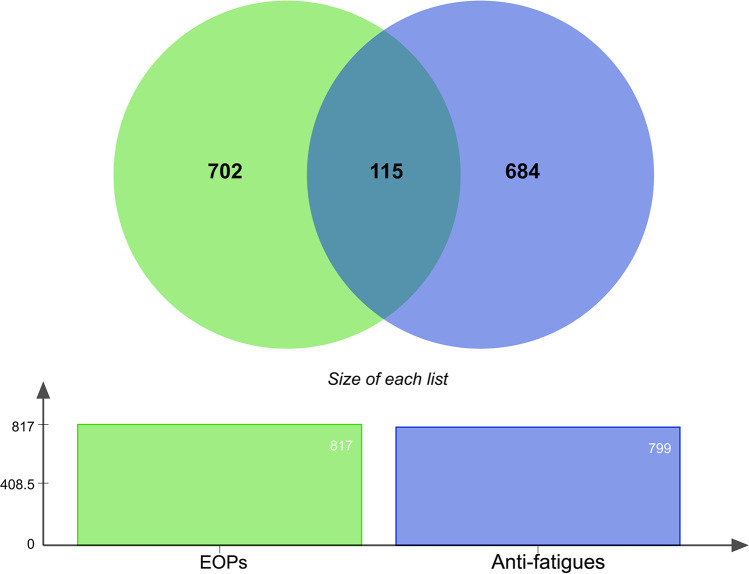
Identifying the potential target for antifatigue.

We comprehensively searched target genes associated with fatigue using GeneCards v5.13, OMIM, and DisGeNET 7.0 databases, identifying over 3,000 fatigue-related targets. Simultaneously, 817 target proteins were predicted from essential oil-derived compounds using SwissTargetPrediction. The intersection of these datasets yielded 115 high-confidence targets for further analysis (Fig 2). The Venn diagram constructed from the 3000 identified genes revealed that 115 potential targets overlapped, suggesting commonality among specific fatigue-related genes. Notably, however, 702 and 684 potential targets did not share overlap, indicating diversity in the genetic pathways associated with fatigue, as shown in Fig 2. These findings highlight diverse potential targets for combating fatigue, with some shared among essential oil plants and others unique to specific genetic pathways. Further exploration of these targets could offer valuable insights into developing effective interventions for managing fatigue, especially the 115 target proteins that share essential oil targets and fatigue-related genes.

Venn diagram of the potential target from essential oil and anti-fatigue target. There are 817 potential targets from crucial oil plants and 799 targets from GeneCards, OMIM, and DisGeNET. 115 targets are intersected with each other. 702 in grey color, not over with antifatigue, and 684 in blue, not intersecting with EOPs.

### Identification of high-potential anti-fatigue compounds from essential oils using a network analysis approach

**Fig 3:**
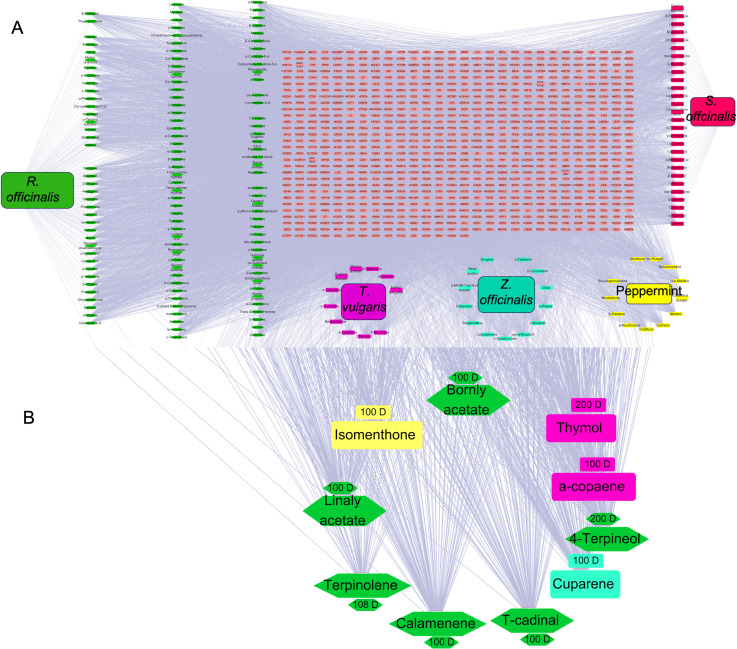
Identification of high-potential anti-fatigue compounds from essential oil plants. (A) The HAT network diagram was employed using Cytoscape 3.10.1. The grey-red diamond denotes potential target proteins of the active compounds from 5 major sources of essential oil plants, which UniProt and SwissTargetPrediction predict. The Five rectangular shapes represent 5 active compounds of *R. officinalis, S. officinalis, T. vulgaris, Z. officinalis*, and Peppermint. Green nodes represent specific compounds extracted from *R. officinalis.* Pink/Magenta nodes represent specific compounds from *T. vulgaris*. Yellow nodes represent specific compounds derived from *Peppermint.* Cyan/Blue nodes denote particular compounds extracted from *Z. officinalis*. (A) The Hub compounds from 5 primary plant sources based on the network are over 100 degrees, with 6 compounds from *R. officinalis*, two from *T. vulgaris*, one from *Z. officinalis*, one from Peppermint, and no compounds from S. o*fficinalis*. These Hub compounds can have the potential for anti-fatigue.

Before that, we performed the HAT network diagram using Cytoscape 3.10.1 to understand the interaction between compound and target proteins, predicted by the SwissTargetPrediction database from *R. officinalis, S, officinalis, T. vulgaris, Z. officinalis,* and Peppermint*.* Network topology analysis revealed novel 888 nodes with 3,103 edges, an average node degree of 6.597, and 189 nodes greater than average (Fig 3A). We identified 10 components among the 125 analyzed with notably high degrees exceeding 100. These compounds include 4-terpineol, ⍺-copaene, bornyl acetate, calamenene, cuparene, isomenthone, linalyl acetate, T-cadinol, terpinolene, and thymol, which are the most active and highest potential for anti-fatigue based on network analysis as shown in Fig 3B. These compounds are likely highlighted because of their strong binding affinities or significant interactions with the target proteins involved in anti-fatigue effects. This network visualization supports the identification of key compounds from essential oils with potential anti-fatigue effects.

### Network analysis reveals key targets in essential oils for anti-fatigue therapy

**Fig 4:**
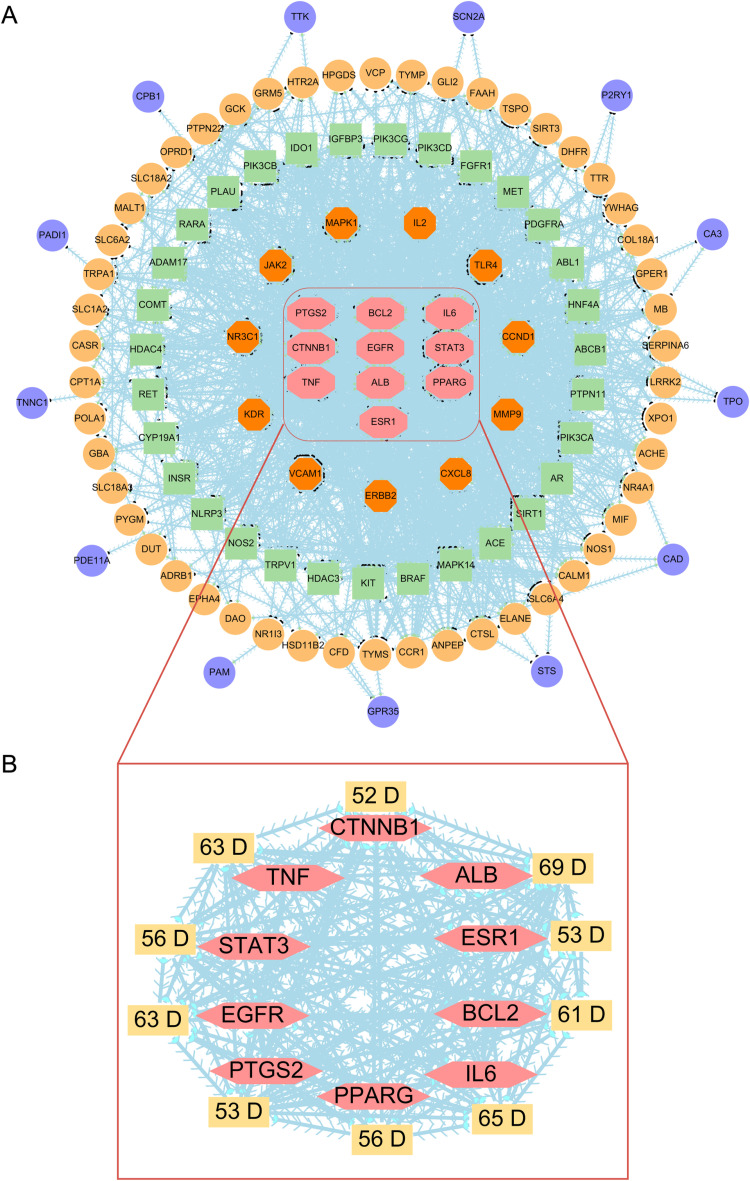
The protein-protein interaction network and the identification of Hub genes. (A) PPI network for anti-fatigue obtained from GeneCards, OMIM, DisGeNET, UniProt, and SwissTargetPrediction, including 115 overlapping proteins from essential oil active compound and target fatigue protein and over 100 interactions. The network present in the circular diagram has different colors and different diameters. The innermost rectangular shapes with grey and red have maximum interaction with over 100 degrees; the orange color circle represents 90–10 degrees; the green circle denotes 50–90 degrees; the grey and yellow circle represents 10–49 degrees; and the purple circle represents 1–9 degrees. (B) PPI revealed 10 proteins that interact with other proteins based on the network topology analysis.

Further, we focus only on 115 overlapping anti-fatigue targets and imported Swiss-predicted target proteins for five major essential oil plants into the STRING 11.5 database to find the new vital target genes for the 10 essential compounds. Network topology analysis revealed that the network comprised 411 nodes and 11,117 edges, with an average node degree of 30. Fig 4A illustrates that nodes closer to the center of the concentric circles are more significant and darker, indicating greater importance using Cytoscape 3.10.1 software. This network revealed 10 essential target proteins which nodes degree over 50 such as albumin (ALB, degree = 69), interleukin-6 (IL6 degree = 65), tumor necrosis factor (TNF degree = 63), epidermal growth factor receptor (EGFR, degrees = 63), B-/lymphoma 2 (BCL2, degree = 61), signal transducer and activator of transcription 3 (STAT3, degree = 56), peroxisome proliferator-activated receptor gamma (PPARG, degree = 56), prostaglandin-endoperoxide synthase 2 (PTGS2, degree = 53), estrogen receptor 1 (ESR1, degree = 53), and Catenin Beta 1 (CTNNB1, degree = 52), were identified as critical targets for fatigue treatment with essential oils (Fig 4B). The close connectivity and extensive interactions among these core proteins, as highlighted in the zoomed-in section, suggest they are critical targets for the bioactive compounds in essential oils for antifatigue.

### GO enrichment and KEGG pathway analysis of essential oil targets reveal critical anti-fatigue pathways

**Fig 5:**
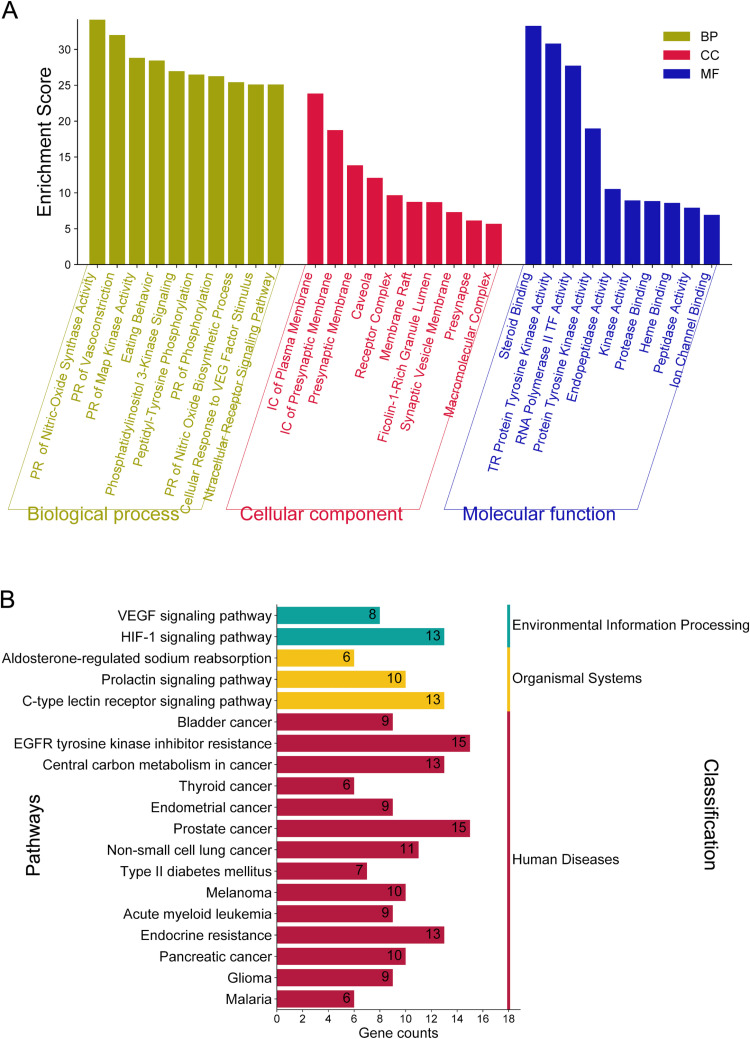
GO enrichment and KEGG pathway analysis of essential oil for anti-fatigue pathways. **(A)** Bar diagram of the GO enrichment analysis. It is performed by the Database for Annotation, Visualization, and Integrated Discovery (DAVID). The bar graph was plotted using the bioinformatics online platform. 10 top of biological process (BP) was selected to analyze their specific pathway, denoted in yellow bar. The red bar represents the ten top Cellular components (CC), and the Molecular function (MF) is denoted in the Blue Bar. The Y-axis represents the enrichment score range from 0 to 60. Each column bar graph represents specific BB, CC, and MF. **(B)** Bar diagram of KEGG pathway analysis. The bar graph was plotted using the bioinformatics online platform by selecting the 20 top KEGG enrichments based on enrichment score. The x-axis represents several genes involved in each pathway. The pathway is classified into three classes: human disease-related pathways are represented in the red bar, the Organismal system pathway is denoted in the yellow bar, and the Environmental information process pathway is denoted in the green bar. The number of each column bar represents the gene counts in the pathway.

To ensure that our selection process did not inadvertently exclude essential pathways, we conducted comparative enrichment analysis between the full-fatigue target set (3,000 + genes) and the final filtered set (115 genes). The GO and KEGG pathway analyses showed that critical pathways involved in inflammatory regulation, oxidative stress, and fatigue-related metabolic dysregulation remained well-represented. Notably, pathways related to neurological differentiation and minor endocrine functions were reduced after filtering, but these were not directly implicated in fatigue regulation. This confirms that our selection process retains biologically relevant targets while minimizing unrelated pathways. We conducted GO enrichment and KEGG pathway analyses using the DAVID v2022q2 database to explore the biological functions and metabolic pathways associated with the 115 overlapping targets. A total of 1,636 GO enrichment entries were obtained, encompassing 1,232 biological process (BP) entries, 145 cellular component (CC) entries, 259 molecular function (MF) entries, and 189 KEGG signaling pathways. The GO enrichment results were visualized through SRplot, with darker colors indicating higher degrees of enrichment. The BP terms were primarily enriched by various biological processing, such as positive regulation of nitric-oxide synthase activity, vasoconstriction regulation, MAP kinase activity, and others. Significantly enriched CC terms included components like the plasma membrane, presynaptic membrane, and receptor complexes. Regarding MF, enrichments were observed in molecular activities such as steroid binding and protein tyrosine kinase activity. Significantly enriched CC terms included Cytosolic ribosomes, large ribosomal subunit, small ribosomal subunit, Polysomal ribosome, Chaperonin-containing T-complex, and NLS-dependent protein nuclear import complex, as shown in Fig 5A. We classified the top 20 enriched KEGG pathways at the pathway level, focusing on those with *p*-values lower than 0.001. Two (VEGF and HIF-1 signaling pathways) were enriched in environmental information processing, while three were highly enriched in the organismal system. Interestingly, the remaining 15 pathways were enriched in metabolic pathways associated with human diseases, indicating their significant role in anti-fatigue processes. Our KEGG pathway analysis identified multiple cancer-related pathways, including thyroid, endometrial, prostate, lung, bladder, and pancreatic cancer, among the top enriched pathways. While these pathways have been associated with fatigue in cancer patients, it is crucial to establish a mechanistic link (Fig 5B). Previous studies have shown that dysregulation of BLC2, ALB, and STAT3 plays a significant role in cancer-related fatigue by driving systemic inflammation and metabolic alterations [43,44]. Similarly, EGFR signaling has been implicated in tumor progression and energy metabolism imbalances contributing to fatigue [45]. However, further experimental studies are necessary to confirm whether the modulation of these targets directly alleviates fatigue symptoms in cancer patients. This finding underscores the complexity of cancer-related fatigue, which tumor-related factors, treatment side effects, and systemic metabolic alterations may influence.

### Comprehensive ADMET evaluation of essential oil-derived compounds for anti-fatigue drug development

Drug discovery and development pose significant challenges and costs, necessitating careful selection, target identification, validation, lead discovery, optimization, and preclinical and clinical trials. To streamline this process, we conducted an ADMET analysis on 10 essential oil-derived compounds to evaluate their drug-likeness and pharmacokinetic properties. Our study included key parameters such as aqueous solubility (logS values ranging from 0 to −6), topological polar surface area (TPSA ≤ 130 Å²), and molecular weight (MW < 500 g/mol). All tested compounds demonstrated moderate solubility and TPSA values below 130 Å², indicating favorable absorption and distribution, with molecular weights supporting drug-likeness. Furthermore, blood-brain barrier (BBB) permeability predictions ranged from 0.88 to 0.99, suggesting solid central nervous system (CNS) accessibility, which is pertinent for fatigue-related neurological conditions (Table 1). While all compounds adhered to Lipinski’s Rule of Five, α-copaene, cuparene, and calamenene surpassed a ClogP of 5, indicating poor aqueous solubility and potential limitations in oral bioavailability (Table 1), underscoring the necessity for formulation strategies such as lipid-based delivery systems or prodrug modifications to enhance their pharmacokinetic properties. Further pharmacokinetic and safety evaluations showed that most compounds exhibited moderate to high plasma protein binding (72–88%), which may influence free drug availability, while CYP metabolism predictions indicated that calamenene and isomenthone might function as CYP enzyme inhibitors (CYP1A2, CYP2C9, CYP2D6), suggesting potential drug-drug interaction risks. Additionally, α-copaene and terpinolene were recognized as CYP3A4 substrates, pointing to rapid metabolism that may reduce their systemic half-life. Toxicity risk assessments indicated that while most compounds exhibited low toxicity, calamenene showed high hepatotoxicity, and terpinolene and cuparene presented potential hERG inhibition, raising concerns about cardiotoxicity (Table 2). These findings emphasize the need for structural modifications or alternative formulations to alleviate toxicity while maintaining biological activity. Based on these results, we performed molecular docking studies on all 10 compounds to evaluate their binding affinity with the target protein, further confirming their potential as antifatigue drug candidates.

ClogP (Lipophilicity): Ideal values for good absorption and bioavailability are generally between 2 and 5.

HBA (Hydrogen Bond Acceptors) and HBD (Hydrogen Bond Donors) influence solubility and permeability. LopS (Lipophilicity Score) indicates overall lipophilicity. BBB (Blood-Brain Barrier Penetration) suggests the ability to cross the blood-brain barrier, which is essential for central nervous system effects. TPSA (topological polar surface area): Lower TPSA can indicate better cell membrane permeability. Plasma Protein Binding (PPB %): Compounds with high PPB (>80%) may have lower free drug availability, which can affect efficacy. CYP Metabolism: Substrates of CYP3A4 and CYP2D6 could have shorter half-lives, while inhibitors may cause drug-drug interactions. Toxicity alerts (hepatotoxicity, mutagenicity, and hERG). Compounds with high PPB may have reduced free drug availability. In contrast, CYP enzyme inhibition or metabolism can affect drug clearance. Toxicity alerts highlight potential risks that may require structural modification or formulation optimization.

### Potent essential oil-derived compounds strongly bind with core target proteins

**Fig 6:**
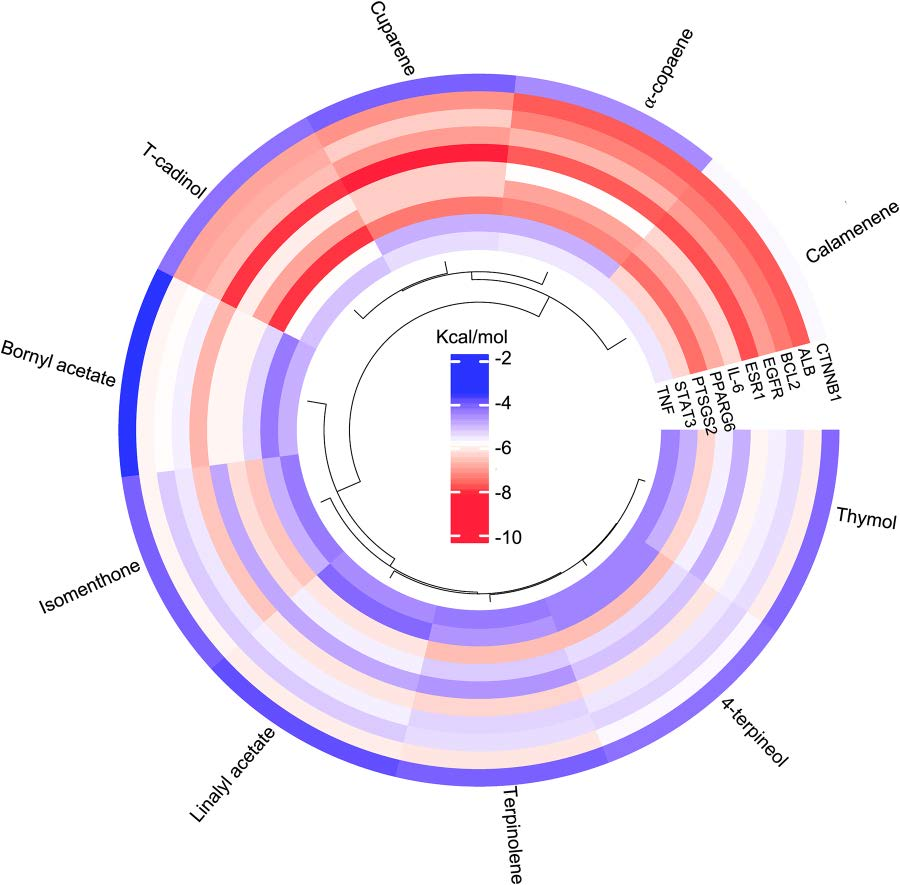
Circular heatmap of the key compounds with core targets.

Furthermore, we employed CB-DocK2 to ascertain the reliability of interactions between essential proteins and critical compounds. The docking results showed that all the key compounds identified using network pharmacology have a moderate affinity for core targets with binding energy ranging from -2 to -10 kcal/mol, as shown in Fig 6, indicating that the docking result also fits the network pharmacology study. Interestingly, calamenene exhibited the strongest binding affinity with all potential targets related to fatigue except TNF, ranging from -6 to -10 kcal/mol. This indicates its ability to interact effectively with various biological and molecular pathways, as shown in Fig 6. This finding highlights calamenene as a promising candidate for further exploration in drug development and therapeutic interventions for fatigue, potentially offering a multifaceted approach to addressing complex physiological processes. Additionally, ⍺-copaene, T-cadinol, and cuparene demonstrated strong binding affinity with ALB, BLC2, EGFR, ESR1, IL-6, PPARG6, PTSG2, and STAT3, as shown in Fig 6 and Table 2. The ability of these compounds to interact effectively with diverse targets underscores their broad pharmacological activity and potential to mitigate fatigue and related processes. To ensure reliability, docking simulations were performed in triplicate for each ligand-protein complex, and the mean docking score ± standard deviation (SD) was calculated (Table 2). The binding affinities of key essential oil-derived compounds were compared with known ligands, such as Warfarin for ALB (-8.2 ± 0.2 kcal/mol) and Venetoclax for BCL2 (-9.0 ± 0.1 kcal/mol). Notably, T-cadinol and α-copaene demonstrated strong binding affinities with ALB (-7.0 ± 0.3 kcal/mol and -7.2 ± 0.2 kcal/mol, respectively), approaching those of the reference ligands, suggesting potential anti-fatigue activity as shown in Table 2. However, α-copaene, cuparene, and calamenene violated Lipinski’s rule of five parameters, especially the ClopP value is over 5, which is shown in Table 1 that led us to examine further the chemical interaction between T-cadinol derived from the essential oil plants with target protein for antifatigue

It represents the binding affinities of various compounds to multiple target proteins measured in kcal/mol. The outermost ring lists the compounds, while the inner rings contain the target proteins. The color scale in the center indicates the binding affinity values, ranging from red (stronger binding, more negative kcal/mol values) to blue (weaker binding, less negative kcal/mol values).

### Potential antifatigue compounds interact with core target protein mainly via hydrogen and hydrophobic interaction

**Fig 7:**
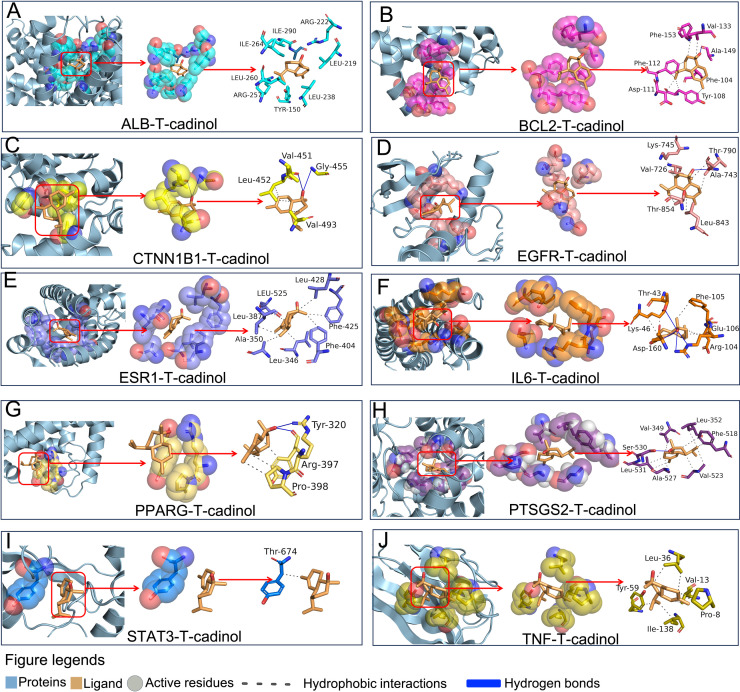
Detailed molecular docking interactions between the compound T-cadinol and various target proteins.

We employed PLIP to characterize the specific chemical interactions between the ligands and their corresponding protein targets to elucidate the molecular interactions. Our analysis revealed that, while the majority of identified compounds primarily engaged in hydrophobic interactions with the target proteins, cuparene demonstrated unique binding characteristics, including π-stacking interactions with Phe residues of BLC2 and π-cation interactions with Lys residues of CTNN1B and IL6, as detailed in 2S Table. Similarly, calamenene formed π-cation interactions with Lys of CTNN1B, which likely contributes to the enhanced binding affinities observed for these compounds. Despite these favorable interactions, cuparene and calamenene were found to violate Lipinski’s rule of five, limiting their potential for clinical application. In contrast, T-cadinol displayed strong binding affinities across multiple target proteins and conformed to Lipinski’s criteria, underscoring its viability as a lead compound for clinical development. Detailed interaction mapping *via* PLIP revealed that T-cadinol engaged in extensive hydrophobic interactions with critical residues such as Tyr-50, Leu-219, and Leu-328 in ALB; Phe-104, Tyr-108, Asp-111, and Phe-112 in BCL2; and Val-726, Ala-743, Lys-745, Thr-790, Leu-844, and Thr-854 in EGFR. Additionally, T-cadinol formed critical hydrogen bonds with residues, including Thr-790 in EGFR, Arg-222 in ALB, and Arg-104 in IL6, further stabilizing these interactions. Moreover, T-cadinol exhibited similar interaction patterns across other target proteins, including PPARG6, PTSGS2, STAT3, and TNF, predominantly through hydrophobic contacts, with selective hydrogen bonding contributing to the overall binding stability. These intricate binding interactions, as shown in Figure 7(A-J), highlight T-cadinol’s potential as a clinically relevant anti-fatigue agent, given its robust binding affinity and compliance with Lipinski’s rule. This suggests a favorable antifatigue for therapeutic use.

Each panel (A-J) represents a different protein that t-cadinol interacts with, highlighting specific amino acid residues and the type of interactions (e.g., hydrophobic interactions, hydrogen bonds) between the ligand (T-cadinol) and target proteins. Detailed visualization of the interaction between the T-cadinol and residues of multiple residues using PLIP from the docking results. The numerous protein residues are represented in stick models. Interactions in the blue line indicate hydrogen bonds between compound and amino acids, the grey dotted line represents hydrophobic interactions, and the PLIP results.

### Validation of docking results via MD simulation with T-cadinol

**Fig 8:**
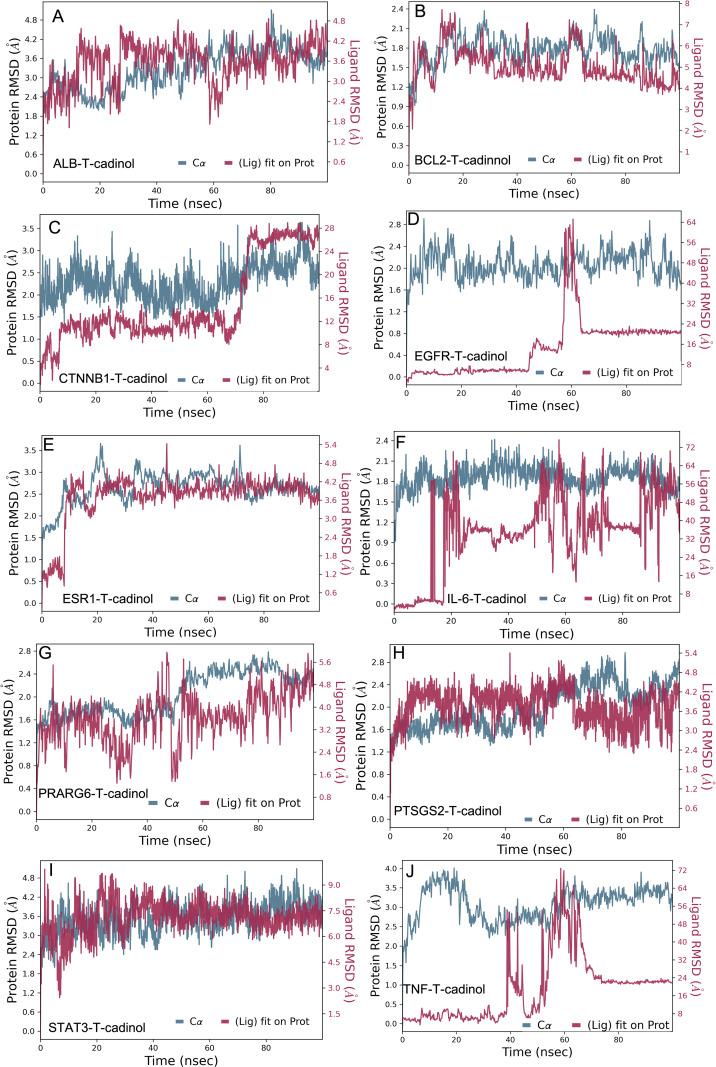
Molecular dynamics (MD) simulation analysis of T-cadinol complexed with key protein targets.

To validate docking results, 100 ns molecular dynamics (MD) simulations were conducted for all ligand–protein complexes, where the docking results were used as the initial states for the simulations. The RMSD value of the complex is mainly focused on the RMSD value of the complex, which is calculated for the protein alpha carbon and protein fit ligand heavy atoms in each complex from the 100 ns MD simulation trajectories. This result confirms the docking predictions, validating the stability and reliability of T-cadinol as a potential anti-fatigue compound. The highly stable complexes (BCL2-T-cadinol, EGFR-T-cadinol, PPARG-T-cadinol, and PTGS2-T-cadinol) demonstrated minimal RMSD fluctuations, maintaining their binding interactions throughout the simulation, as shown in Fig 8D, D, G, and H. These findings reinforce that these targets provide favorable binding environments for T-cadinol, confirming strong molecular docking predictions. The moderately stable complexes (ALB, ESR1, and STAT3-T-cardinal) exhibited minor flexibility (Fig 8A, E, and I). Yet, these retained their interactions with acceptable fluctuations, indicating that additional validation through in vitro and in vivo studies is necessary while the binding is present. Conversely, CTNNB1-T-cadinol, IL-6-T-cadinol, and TNF-T-cadinol complexes showed instability, marked by significant ligand RMSD deviations, suggesting weak or transient binding interactions as shown in Fig 8C, F, and J. This discrepancy between docking and MD results highlights the necessity of MD simulations as a refinement step, ensuring only truly stable interactions progress to experimental validation. Ultimately, integrating molecular docking with MD simulations refines the selection of bioactive compounds, offering a powerful approach to computational drug discovery. These findings support the continued exploration of T-cadinol as a potential therapeutic for fatigue-related conditions, paving the way for future preclinical and clinical assessments.

Root Mean Square Deviation (RMSD) plots for protein (Cα backbone) and ligand (T-cadinol) over a 100 ns MD simulation. Protein RMSD is shown in blue, while ligand RMSD (relative to the protein) is in red. These analyses assess the stability and conformational fluctuations of T-cadinol-bound complexes with various fatigue-related target proteins. Each panel (A-J) represents a different protein with T-cadinol for MD simulation over 100 ns.

## Discussion

Fatigue is a well-documented symptom in cancer patients, often associated with systemic inflammation, metabolic dysfunction, and treatment-induced stress [1,2]. Understanding the mechanisms underlying fatigue, mainly its association with various cancers that affect the nervous system and factors such as mitochondrial dysfunction, immune activation, and neurotransmitter imbalance [6,7]. Moreover, it underscores the potential of natural compounds, such as those derived from plants and fungi, in mitigating fatigue [46] and herbal medicines and nutraceuticals known for their anti-fatigue effect. However, there are challenges in developing and utilizing essential oil derivative compounds due to their complex composition and unclear product placement. Essential oils exhibit diverse biological activities and exert their effects through the olfactory and central nervous systems [20]. This study proposes using network pharmacology and molecular docking to investigate the potential anti-fatigue properties of essential oil compounds derived from five primary plant sources: *R. officinalis, T. vulgaris, S. officinalis, Z. officinalis,* and Peppermint. These computation methods emphasize efficiency and predictive accuracy in drug discovery and mechanism elucidation.

This study comprehensively analyzes 872 essential oil derivative compounds sourced from five major plants: *R. officinalis, T. vulgaris,* Peppermint*, S. officinalis,* and *Z. officinale*, known for their diverse therapeutic properties. The analysis revealed a substantial compound overlap across these sources, with 125 compounds intersecting among all plants, suggesting both shared and unique chemical constituents. Using computational Evenn, potential target proteins for these compounds were predicted, indicating many shared possible targets among the plants. This implies that, despite the botanical diversity, certain bioactive compounds in these plants may exert their therapeutic effects through similar molecular targets or pathways, such as stress, as fatigue is also associated with cancer. In addition, bornyl acetate has been shown to have stimulating and mood-enhancing effects, providing a natural energy boost and combating feelings of lethargy [47]. More importantly, 4-terpineol, thymol, linalyl acetate, T-cadinol, and bornyl acetate are derived from *R. officinalis,* commonly used for mental strain and fatigue in folk medicine [48]. Peppermints contain isomenthone, which is robust in relieving exercise-induced fatigue [49]. Moreover, cuparene, derived from *Z. officinale*, exhibited anti-inflammatory activity by inhibiting the NF-κB-activated pathway in a dose-dependent might explain why these compounds have more potential for anti-fatigue. Interestingly, ⍺-copaene is a component of *T. vulgaris* that has not been well studied regarding anticancer or fatigue. Therefore, our study presents new potential compounds for anti-fatigue.

Our study shows that new target proteins involved in cancer and inflammatory responses, such as CTNNB1, become a biomarker for exercise anticancer effects in colorectal cancer and contribute to skeletal myopathy in heart failure via direct interaction FoxO [50]. EGFR is vital in cell survival, proliferation, stress-induced trafficking, autophagy, and energy metabolism imbalances, affecting cellular health and recovery and influencing fatigue [51]. This suggests that EGFR can be a new target for treating fatigue. IL-6 plays a dual role in inflammation and immune response and activates the STAT3, significantly contributing to autoimmune and inflammatory diseases such as rheumatoid arthritis [52], indicating that elevated levels are often associated with chronic fatigue, especially in autoimmune and inflammatory diseases. While our study identifies 115 core targets associated with essential oil-derived compounds for antifatigue, we acknowledge potential selection bias in the target identification process. We compared enrichment results for the complete set of 3,000 + fatigue-related genes versus the final 115 high-confidence targets to assess whether critical pathways were lost due to filtering. This analysis confirmed that key pathways, including inflammatory response, metabolic regulation, and oxidative stress, remained well-represented, ensuring our results reflect biologically relevant fatigue mechanisms. The GO enrichment and KEGG pathway analysis notably revealed that the enriched pathways were predominantly associated with metabolic pathways linked to human diseases, particularly cancer types such as thyroid, endometrial, prostate, lung, bladder, and pancreatic cancer. Our study identified core genes (ALB, BCL2, EGFR, IL-6, and STAT3) enriched in cancer pathways, suggesting a potential link between cancer-related metabolic dysregulation and fatigue. IL-6 and STAT3, in particular, have been experimentally linked to fatigue in cancer patients due to their roles in cytokine signaling and mitochondrial dysfunction [53]. Additionally, evidence from previous studies has shown that EGFR inhibition may improve energy metabolism and reduce fatigue symptoms [54]. This study suggests a strong association between cancer-related fatigue and the dysregulation of metabolic processes, implicating advanced cancer stages in fatigue development. The identification of these pathways underscores the complexity of cancer-related fatigue, influenced by tumor-related factors, treatment side effects, and systemic metabolic alterations. However, our findings are based on computational analysis, and further in vitro and in vivo studies are required to validate these associations. While these processes may contribute to fatigue in specific conditions, their exclusion is unlikely to impact our core findings significantly.

Additionally, we have expanded our discussion to acknowledge the false positive/negative rates inherent in SwissTargetPrediction and other bioinformatics tools. To mitigate these risks, we validated key interactions using CB-Dock2 docking, PLIP interaction profiling, and MD simulation. Furthermore, molecular docking revealed that calamenene binds with the strongest binding affinity across all potential targets, suggesting its efficacy in combating fatigue. It also fits the point that inhalation of calamenene powerfully relieves exercise-induced fatigue. Conversely, ⍺-copaene, T-cadinol, and cuparene have displayed a moderate binding affinity with several targets but a strong affinity with others. More interestingly, calamenene shows the highest binding affinity with CTNNB1, which might be due to forming π-cation interactions with Lys of CTNN1B, which likely contributes to the enhancement of binding energies due to cation-Pi interactions between aromatic amino acids and organic or metal ions [55]. It occurs due to the presence of the aromatic ring in compounds or target proteins, such as π-stacking interactions with Phe residues of BLC2 with cuparene [55], which provide good interaction with the target protein to be antifatigue.

Despite their strong target binding affinities, α-copaene, cuparene, and calamenene exceed Lipinski’s rule ClogP threshold of 5, suggesting potential limitations in oral absorption, aqueous solubility, and high toxicity [56]. However, Lipinski’s rule is primarily designed for small-molecule oral drugs and does not necessarily exclude these compounds from therapeutic applications. Several strategies could be explored to enhance their drug-likeness while maintaining activity which including the minor modifications, such as introducing polar functional groups or prodrug synthesis, could help reduce excessive lipophilicity without compromising binding efficacy, nanoemulsions, liposomes, or solid lipid nanoparticles (SLNs) could improve solubility and increase bioavailability via lymphatic absorption, bypassing first-pass metabolism [57], enhancing solubility and stability of lipophilic compounds by forming water-soluble inclusion complexes [58], and combining lipid carriers with polymeric nanoparticles may provide a dual mechanism for improving stability and controlled release [59]. These efforts could bridge the gap between their potent bioactivity and limitations in drug-likeness. Interestingly, we found that T-cadinol interacted more with multiple target proteins that involve hydrophobic interaction with ALB, BCL2, and EGFR via hydrophobic interaction and hydrogen bonds with residues, including Thr-790 in EGFR, Arg-222 in ALB, and Arg-104 in IL6. It also revealed that Tyr-108, similar to Tyr-50 in ALB, provides additional stability through hydrogen-bonding interactions, contributing to the overall binding energy [60]. More importantly, inflammatory cytokines disrupt normal metabolic processes and neurotransmitter balance, contributing to the sensation of fatigue [61]. T-cadinol has been demonstrated to modulate inflammatory pathways, which could reduce systemic inflammation [62]. T-cadinol demonstrated a high binding affinity to inflammation- and metabolism-related proteins, suggesting potential benefits in chronic fatigue syndrome (CFS), cancer-related fatigue (CRF), and inflammatory fatigue disorders [63]. Additionally, ADMET analysis confirmed that T-cadinol exhibits good drug-likeness, with high oral bioavailability, metabolic stability, and minimal toxicity risks, making it a strong candidate for therapeutic development. Its ability to cross biological membranes, including potential blood-brain barrier permeability, indicates possible neurological benefits, which could help counteract fatigue-related cognitive dysfunction. Similarly, MD simulation demonstrates that T-cadinol has stable interaction with ALB, BCL2, EGFR, and STAT3. Fatigue treatments include stimulants (e.g., modafinil, caffeine) and adaptogens (e.g., ginseng, rhodiola) [64,65]. Still, many of these have side effects, short durations of action, or limited efficacy. Unlike synthetic stimulants, T-cadinol and other essential oil-derived compounds may offer natural, multi-targeted therapeutic potential with fewer adverse effects. Given its antioxidant, anti-inflammatory, and neuroprotective properties, T-cadinol could be a safer alternative or adjunct therapy for fatigue management.

Despite promising in silico findings, experimental validation is essential to confirm T-cadinol’s pharmacological effects. Future studies should focus on in vitro and in vivo models to assess its bioavailability, metabolism, and therapeutic efficacy in fatigue-related conditions. Additionally, clinical trials could evaluate its effectiveness in reducing fatigue symptoms compared to existing treatments. Other compounds identified in this study with favorable ADMET profiles, such as α-copaene, cuparene, and calamenene, should also be further investigated for their potential synergistic effects in multi-compound fatigue therapies. The docking scores of the identified essential oil-derived compounds were evaluated against well-characterized reference ligands for their respective targets. The binding affinities of α-copaene (-7.8 kcal/mol) and T-cadinol (-7.0 kcal/mol) to ALB and BCL2 were comparable to those of standard inhibitors (-8.2 and -9.0 kcal/mol, respectively), indicating their potential as antifatigue agents. However, further experimental validation must confirm their biological relevance, including in vitro and *in vivo* assay. This study provides a scientific foundation for developing essential oil-derived compounds as novel antifatigue therapeutics by integrating network pharmacology, molecular docking, ADMET analysis, and MD simulation.

## Conclusion

This study is the first to integrate network pharmacology, molecular docking, and molecular dynamics (MD) simulations to systematically identify specific compounds derived from essential oils that have potential antifatigue effects by targeting key genes associated with fatigue. Among the identified bioactive compounds, T-cadinol showed the most significant interactions with ALB, BCL2, STAT3, and IL-6, forming stable hydrophobic and hydrogen bond interactions, as confirmed by 100 ns MD simulations showing favorable ADMET profiles. Although other essential oil compounds, such as bornyl acetate, calamenene, thymol, α-copaene, and cuparene, have less favorable ADMET profiles, they may influence fatigue-related pathways and offer therapeutic potential for managing fatigue and metabolic dysregulation. Furthermore, this study highlights a potential link between fatigue-related targets and cancer-related pathways, suggesting that modulating these core proteins may help alleviate fatigue and reduce the risk of cancer progression in conditions such as thyroid, endometrial, prostate, lung, bladder, and pancreatic cancers. While our *in silico* findings provide a strong foundation for understanding the mechanisms of essential oil-derived compounds, further in vitro and in vivo studies are needed to validate their biological efficacy, pharmacokinetics, and safety. This research emphasizes the increasing role of computational methods in speeding up natural product-based drug discovery, providing a cost-effective, high-throughput approach to identifying novel antifatigue agents. Integrating network pharmacology, cheminformatics, and molecular modeling offers a structured framework for uncovering multi-targeted, mechanism-based therapeutics. Future advancements in AI-driven virtual screening and machine learning-based molecular simulations will improve the precision, scalability, and clinical translation of computational drug discovery. These findings establish a foundation for future experimental validation and potential therapeutic applications of essential oil-derived compounds in fatigue management.

## Supporting information

S1 Table Detailed sources of target proteins and compounds from 5 primary essential plants. (XLSX)

S2 Table Molecular docking interactions of various compounds with the target protein. (XLSX)

S3 Table Distribution of essential oil-derived compounds across different plant species. (DOCX)
