# Supplementary material for: Essential oil-derived compounds target core fatigue-related genes: A network pharmacology and molecular Docking approach
Source: PLoS One. 2025 May 28;20(5):e0314125. doi: 10.1371/journal.pone.0314125 (PMC12118864; doi:10.1371/journal.pone.0314125)
Supplement: S3 Table — (DOCX) [file pone.0314125.s003.docx]

**Table S3 Shared and unique compounds across analyzed plants**

| **Compound  Name** | **Rosmarinus  officinalis** | **Salvia  officinalis** | **Thymus  vulgaris** | **Zingiber  officinale** | **Mentha × piperita** | **Shared/Unique** |
| --- | --- | --- | --- | --- | --- | --- |
| 4-terpineol | ✓ | ✓ |  |  |  | Shared (R. officinalis, S. officinalis) |
| α-copaene | ✓ | ✓ | ✓ |  |  | Shared (R. officinalis, S. officinalis, T. vulgaris) |
| Bornyl acetate |  |  | ✓ | ✓ |  | Shared (T. vulgaris, Z. officinale) |
| Calamenene | ✓ |  |  |  |  | Unique to R. officinalis |
| Cuparene |  | ✓ |  |  | ✓ | Shared (S. officinalis, M. × piperita) |
| Isomenthone |  |  | ✓ | ✓ | ✓ | Shared (T. vulgaris, Z. officinale, M. × piperita) |
| Linalyl acetate |  |  | ✓ |  |  | Unique to T. vulgaris |
| T-cadinol | ✓ | ✓ | ✓ | ✓ | ✓ | Shared (All plants) |
| Terpinolene |  |  |  | ✓ |  | Unique to Z. officinale |
| Thymol |  | ✓ | ✓ |  |  | Shared (S. officinalis, T. vulgaris) |

This table presents the distribution of essential oil-derived compounds across different plant sources. Shared compounds are indicated with (✓), while unique compounds to a specific plant are marked accordingly. The Shared/Unique column specifies if the compound is shared among multiple plants or unique to one plant. The table is based on the essential oil-derived compounds analyzed in the study.
